# Integrative snRNA‐seq, molecular docking and dynamics simulations identifies Lasmiditan as drug candidate for Alzheimer's disease

**DOI:** 10.1002/ctm2.70443

**Published:** 2025-08-15

**Authors:** Martin Nwadiugwu, Md Selim Reza, Boluwatife Afolabi, Demetrius M. Maraganore, Hui Shen, Hongwen Deng

**Affiliations:** ^1^ Tulane Center for Biomedical Informatics and Genomics, Deming Department of Medicine Tulane University School of Medicine, Tulane University New Orleans Louisiana USA; ^2^ Department of Neurology, Center for Clinical Neurosciences Tulane University School of Medicine, Tulane University New Orleans Louisiana USA

**Keywords:** Alzheimer's disease, drug repurposing, gene regulators, Lasmiditan, snRNA‐Seq

## Abstract

**Background:**

Alzheimer's disease (AD) is a growing healthcare crisis with limited effective therapies. This study aims to identify new candidate drugs that can be repurposed using key transcriptional regulators (DERs) in AD as therapeutic targets.

**Methods:**

Multi‐cohort single‐nucleus RNA sequencing (snRNA‐seq) data from the prefrontal cortex were analysed to identify DERs. Molecular docking and dynamic simulations analysis evaluated interactions between DERs and 2200 Food and Drug Administration‐approved drugs to assess binding stability, whilst pharmacokinetic parameters relevant to blood–brain barrier permeability were evaluated.

**Results:**

We identified 20 key DERs associated with AD. Lasmiditan stood out as the most promising drug amongst other drug candidates (Vorapaxar, Bictegravir, Tonaftate, Fluspirilene, Lisuride, Olaparib) interacting with five DERs: ZEB2, APP, PAX6, ETV6, and ST18. Lasmiditan–ETV6 complex showed the best binding stability (RMSD: 2.98 Å, H‐bonds: 68.38) and optimal passive diffusion (Log*P*3–4, TPSA 60–75 Å^2^).

**Discussion:**

Lasmiditan is a potential AD therapeutic candidate that warrants further preclinical validation.

**Key points:**

- 20 key transcriptional regulators (DERs) were identified linked to AD in myeloid, and neuronal cell populations. The DERs correlated with Braak stage, APOE genotype, and aging.
- ETV6 is a potentially viable therapeutic target due to its ability to form stable and strongly interacting complexes across multiple drugs.
- Lasmiditan showed the strongest binding to ETV6 (RMSD: 2.98 Å, H‐bonds: 68.38) and optimal blood‐brain‐barrier (BBB) penetration (Log*P* 3–4, TPSA 60–75).
- Lasmiditan is a potentially promising AD therapeutic candidate that warrants further preclinical validation.

## Graphical abstract

Multi‐cohort snRNA‐seq analysis identified 20 key transcriptional regulators in Alzheimer's disease (AD). Lasmiditan was the top drug identified for repurposing as it showed strong binding stability with ETV6 and favourable blood–brain barrier permeability.

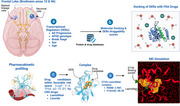

## 1 BACKGROUND

Alzheimer's disease (AD) is a complex neurodegenerative disorder with a significant unmet medical need; a person in the United States develops AD every 65 seconds,^1^, ^2^ and disparities exist as nearly two‐thirds of Americans with AD are women.^2^ Whilst AD diagnosis has recently advanced significantly with the advent of highly accurate blood biomarkers that detect amyloid positivity with ∼95% accuracy, and increased access to amyloid Positron Emission Tomography (PET) scans,^3^, ^4^ the therapeutic landscape remains challenging. Currently, two disease‐modifying therapies, lecanemab and donanemab, have received Food and Drug Administration (FDA) approval; but their impact is constrained by modest efficacy, appreciable adverse effects and significant cost.^5^, ^6^ The paucity of diverse and effective therapeutic interventions for AD persists, and is contributing to a growing healthcare crisis.^7^, ^8^, ^9^, ^10^ For instance, whilst there are over 1500 active immunotherapy clinical trials for drug development in cancer,^11^ only 164 trials are assessing 127 drugs in the 2024 AD drug development pipeline.^12^ This indicates a considerable gap in AD drug development, which is reported to have a failure rate of 99.6%.^12^

Indeed, our understanding of the molecular and cellular mechanisms underlying AD is rapidly evolving; however, translating these insights into effective therapies remains a major challenge. To address this critical need, we present a comprehensive framework for AD drug repurposing, designed to accelerate the translation of basic research to therapeutic application. Given the limited number of repurposed AD therapies (∼40),^13^ we introduce an integrative pipeline to systematically identify and validate key transcriptional regulators driving AD pathogenesis. Using multi‐cohort single‐nucleus RNA sequencing (snRNA‐seq) data from the prefrontal cortex (PFC), we construct gene regulatory networks to identify differentially expressed master regulators (DERs) that will serve as targets for our in silico drug repurposing pipeline. This in silico analysis involves molecular docking of approved drugs from the FDA to DER targets, followed by molecular dynamics (MD) simulations to validate their stability, and predict the binding affinity of the top‐ranked drug‐DER complexes. Whilst DERs or transcriptional factors (TFs) have traditionally been considered undruggable due to lack of active enzymatic sites, recent evidence indicates that certain DERs possess ligandable pockets suitable for small‐molecule modulation.^14^, ^15^ Also, advances in computational structural modelling and fragment‐based screening have enabled the identification of TF binding sites, and led to the development of TF‐targeting compounds which supports the potential for direct drug–TF interactions.^14^

We will predict the druggability of DERs, and utilise molecular docking for drug repurposing to predict binding affinities and interaction modes between drugs and biological target.^16^ Molecular docking is a method that aids in identifying new therapeutic uses for existing drugs by providing structural insights into protein–ligand interactions, evaluating multitarget potential, and offering deeper understanding of drug mechanisms and their expanded applications.^17^, ^18^ Our study will employ snRNA‐Seq data to identify novel transcriptional targets; which would be used for our molecular docking and simulation analysis to identify potential drugs that can be repurposed. Next, pharmacokinetic profiling will be carried out to ensure the selected drugs are relevant to the central nervous system (CNS). Our pipeline will hopefully accelerate the transition from basic molecular insights to therapeutic applications and enhance the efficacy of repurposed drugs for AD.

## 2 MATERIAL AND METHODS

### 2.1 Drug screening by molecular docking

This study adopted molecular docking to perform the interaction analysis between the target DERs and drug ligand molecules. We collected 2200 approved FDA drugs from DrugBank database (DrugBank^19^ v6.0, https://go.drugbank.com) to explore effective drugs for AD (see Table S1). The docking analysis was performed between 23 target DERs and 2200 approved drugs. The three‐dimensional (3D) structures of the targets were obtained from the Protein Data Bank (PDB) (https://www.rcsb.org/). In cases where the 3D structures were unavailable in the PDB, the AlphaFold (AF) models were downloaded from UniProt (https://www.uniprot.org/). To evaluate the druggability of identified DERs, we used the 3D Protein structures or Uniprot ID in DoGSiteScorer (https://proteins.plus) to predict and rank their ligand‐binding pockets. DERs with high druggability were indicated by a Drug Score above 0.7. We utilised the “Discovery Studio Visualizer” to visualise the 3D structures of protein interfaces (https://www.3ds.com/products/biovia). PDB2PQR and H++ servers were used to assign the protonation state of the target proteins.^20^, ^21^ All the missing hydrogen atoms were also appropriately added. The *pKa* for target proteins residues were investigated under the physical conditions of salinity = 0.15, internal dielectric = 10, pH = 7, and external dielectric = 80. On the other hand, the drugs were minimised energy through Avogadro.^22^ For docking, the protein binding sites were defined either based on the location of co‐crystallised ligands (when available) or predicted using COACH‐D v2.0, an accurate deep‐learning‐based binding pocket predictor.^23^ The target proteins were solvated with water, and only polar hydrogens were added. Grid boxes were constructed using AutoDock Tools 4.2 based on the predicted or co‐crystallised binding sites, ensuring that all key binding‐site residues (i.e., interacting amino acids) were completely enclosed within the grid boundaries, and the pdbqt files of proteins were generated.^24^ Similarly, the drug agents were prepared with default parameters, and only Gasteiger charges were added. Subsequently, molecular docking between DERs and the drugs were performed to calculate their binding affinities (kcal/mol) by using AutoDock Vina.^25^

Flexible ligand docking was performed applying the Lamarckian Genetic Algorithm with an exhaustiveness value of 8. After the docked protein–ligand complexes were created, the binding sites were analysed to construct a 2D representation of the ligand interaction for each complex. The protein–ligand complexes were further visualised in Discovery Studio Visualizer and PyMol(https://pymol.org/). Then, we selected the top‐ranked drugs based on the docking score for further analysis.

### 2.2 Molecular dynamics simulation for top complexes

MD simulations assess the stability, flexibility, and dynamic behaviour of drug–target complexes within a simulated biological environment.^26^ This step is crucial for verifying the docking results by simulating the complex's movement over time, which helps evaluate its stability and potential conformational changes. Additionally, MD simulations can explore the effects of mutations in AD‐related proteins, ensuring that the drug remains effective under various biological conditions. Therefore, we performed MD simulations using Nanoscale Molecular Dynamics (NAMD) to study the top DER–ligand complexes.^27^ The DER–ligand complexes were prepared by merging their topology and coordinate files using visual molecular dynamics (VMD)’s psfgen plugin. The ligands were parameterised via CHARMM‐GUI's Ligand Reader & Modeler, and DER proteins were obtained from the PDB. The merged systems were solvated in a Transferable Intermolecular Potential 3P (TIP3P) water box and neutralised with counterions. Equilibration was performed at set temperature of 303.15 K using Langevin dynamics and Nose–Hoover Langevin piston pressure control. The initial complexes were prepared in VMD and CHARMM‐GUI Solution Builder,^28^ and the production simulations run for 100 nanoseconds (ns) with periodic boundary conditions using the CHARMM36m force field and associated toppar files. A timestep of 2 picoseconds (ps) was maintained throughout the simulation. Long‐range electrostatics were handled using particle mesh Ewald (PME) electrostatics with a grid spacing of 1.0 Å, and van der Waals interactions cutoff was at 12 Å. The root mean square error (RMSD) and the solvent‐accessible surface area (SASA) for both the DER and the ligand was computed using the Shrake–Rupley algorithm^29^ implemented in MDTraj^30^ to quantify the extent of their interaction interface. The RMSD is recorded at regular intervals to capture conformational changes and determine the equilibrium state of the complex.

Hydrogen bond (H‐bond) analysis was performed using the Wernet–Nilsson method in MDTraj (v1.9.7)^30^ with a distance cutoff of 0.35 nm and an angle cutoff of 120° to determine the number of hydrogen bonds formed between the drug and the DER targets, throughout the simulation. The DER and ligand were defined by selecting atom groups in the protein structure file (PSF), with the DER designated as all ‘protein’ atoms and the ligand selected by its residue name (‘LIG’). The DER–ligand complexes were analysed with MDAnalysis (v2.4.2) for 100 ns MD simulation. For each complex, statistical analyses of the mean and standard deviation were calculated to provide a quantitative measure of stability and binding affinity; the time‐dependent data were visualised using Matplotlib (v3.7.1). Post MD simulation analysis results were cross‐validated using atom selection consistency checks and trajectory slicing to minimise artefacts.

### 2.3 Predicting blood–brain barrier permeability

We retrieved SMILES representations of selected drugs from ChEMBL database^31^ using RDKit^10^, ^32^ and computed the molecular descriptors, including physicochemical properties such as Log*P* (octanol–water partition coefficient to measure lipohilicity), log*D* (the distribution coefficient of a molecule between an aqueous and lipophilic phase), topological polar surface area (TPSA), and molecular weight (MW). Blood–brain barrier (BBB) permeability was estimated using logBB (blood–brain barrier partition coefficient) with these classification threshold (very high ≥ 0.7, high < 0.7 ≥ 0.3, moderate < 0.3 ≥ −0.3, low ← 0.3 ≥ −0.7, very low < −0.7). We computed logBB using an empirical equation derived from Clark's model^33^: logBB = 0.152 × Log*P* − 0.0148 × TPSA + 0.139, which has been widely used for BBB permeability predictions.^33^ Additionally, P‐glycoprotein (P‐gp) efflux potential was predicted using the threshold: Log*P* > 4 and TPSA < 75. These pharmacokinetic parameters (LogBB, TPSA, MW, Log*P*, Log*D*) were used to evaluate BBB permeability and neurotherapeutic potential of the drugs.

### 2.4 Identification of the transcriptional targets

To investigate master regulators in AD across Brodmann areas 10 (BA10) and 46 (BA46) of the PFC, we leveraged publicly available snRNA‐seq datasets from Anderson et al,^34^ UCI AD Multiome^35^ and ROSMAP^36^ cohorts. The UCI data were sampled from the PFC but were not specific to any BA; and primarily served as validation for data from the ROSMAP (BA10) and Anderson cohorts (BA46). Data from Anderson et al.^34^ were sourced from NIH Neurobiobank, the Human Brain and Spinal Fluid Resource Center, and the Pritzker Neuropsychiatric Disorders Research Consortium. UCI AD Multiome data^35^ was obtained from the UCI MIND's Alzheimer's Disease Research Center (ADRC), whilst ROSMAP^36^ data were accessed from the Religious Orders Study and Memory and Aging Project. Across all datasets, we analysed 81 samples consisting of 42 AD and 39 control cases. The ROSMAP^36^ cohort included 24 AD and 24 control samples evenly split by sex, with 46 Non Hispanic Whites (NHWs) and two African Americans. Anderson et al.^34^ cohort consisted of 7 AD and 8 control samples, primarily from BA46 and were all NHWs, whilst UCI AD Multiome^35^ dataset had 11 AD and 7 control samples. The UCI AD Multiome^35^ dataset had no data on race/ethnicity. Information on the sample preparation steps, IRB approvals and single nuclei extraction protocols for each data cohort can be found in their respective studies.^34^, ^35^, ^36^

To ensure high‐quality data, cells with fewer than 800 UMIs or 500 detected genes were excluded. The datasets were normalised and batch effects corrected using Seurat v4.0.^37^ Cell types were then inferred using SingleR^38^ with reference datasets from Blueprint^39^ and ENCODE,^40^ and we subsequently focused on cells with neuronal and myeloid lineages for further downstream analysis. Metacells were generated to aggregate similar cells of these cell types based on distance metrics derived from PCA embeddings across similar cell clusters (using a minimum of 1000 cells per cluster).^41^ To construct regulons, which forms the regulatory networks that define interactions between the master regulators or transcription factors (TFs) and their target genes, we used the Algorithm for the Reconstruction of Accurate Cellular Networks (ARACNe‐AP) on log2‐counts‐per‐million (log2‐CPM)‐normalised metacell matrices with a mutual information threshold of 0.05, data processing inequality tolerance of 0.05, and 100 bootstraps. This yielded the top 50 TF–target interactions per regulon. MetaVIPER (Virtual Inference of Protein‐activity by Enriched Regulon analysis) was then used to infer regulon activity,^42^ computing *z*‐transformed activity scores. The top 50 regulon interactions were pruned to retain the most significant ones, and their profiles were clustered and visualised using Uniform Manifold Approximation and Projection (UMAP) dimension reduction technique.^43^ The clustering solutions were optimised using Louvain,^44^ and Silhouette Scores were calculated to assess cluster quality.^45^

**FIGURE 1:**
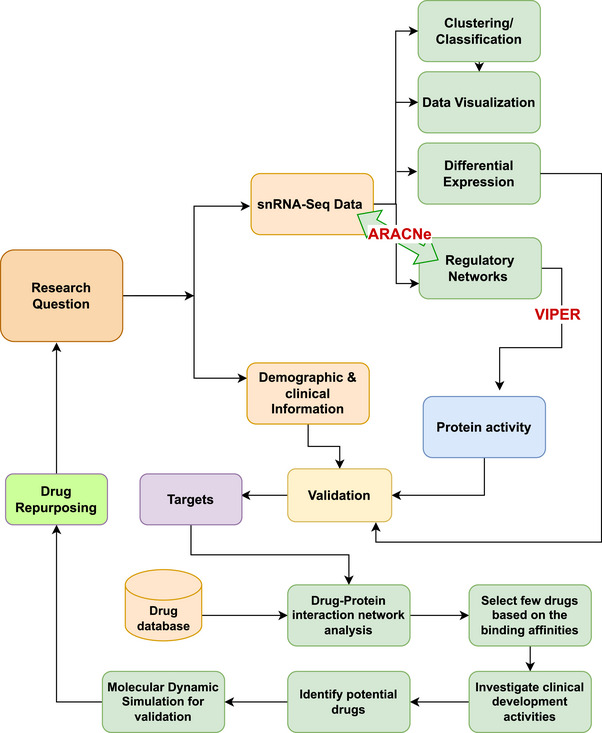
Integrative pipeline for drug repurposing.

For identifying DERs, we applied Stouffer's method.^46^ This method integrates *p*‐values to account for cluster effects and generates association scores to classify regulators as upregulated or downregulated.^47^ Regulons were considered significantly active if they passed a Benjamini–Hochberg (BH)‐adjusted false discovery rate (FDR) threshold of *p* < 0.05 and had an absolute association score > 1.5. Network interaction analysis of the DERs was carried out using IPA software and statistical analyses were performed using R Studio version 4.2.2.^48^ We validated BA10 and BA46‐specific regulators using UCI multiome AD data and the ChIP‐X Enrichment Analysis Version 3 (ChEA3) web tool^49^; our integrated analysis pipeline is shown in Figure 1.

## 3 RESULTS

Molecular docking and virtual screening are speedy, cost‐effective, and reliable methods for finding a possible druggable target well suited for a drug through computer‐aided drug design (CADD).^50^ CADD is currently employed to swiftly annotate and assess vast pharmacological libraries. Figure 1 outlines our systematic approach to drug repurposing. Using multi‐cohort snRNA‐seq data from Anderson et al.^34^ and ROSMAP,^36^ we analysed transcriptional expression patterns in two key brain areas (Brodmann areas 10 and 46), considering factors like age, sex, and clinicopathological features of AD. We constructed gene regulatory networks using ARACNe‐AP algorithm,^51^ and applied metaVIPER^42^ to predict transcriptional activity. The key differences in the transcriptional activity were identified using Stouffer's method, and we confirmed our findings using additional independent data from the UCI AD Multiome^35^ study. The validated DERs were used as targets in drug–DER interaction analyses to identify candidate drugs from DrugBank database. DoGSiteScorer was used to identify highly druggable pockets in the target DERs.

### 3.1 The target regulators

**FIGURE 2:**
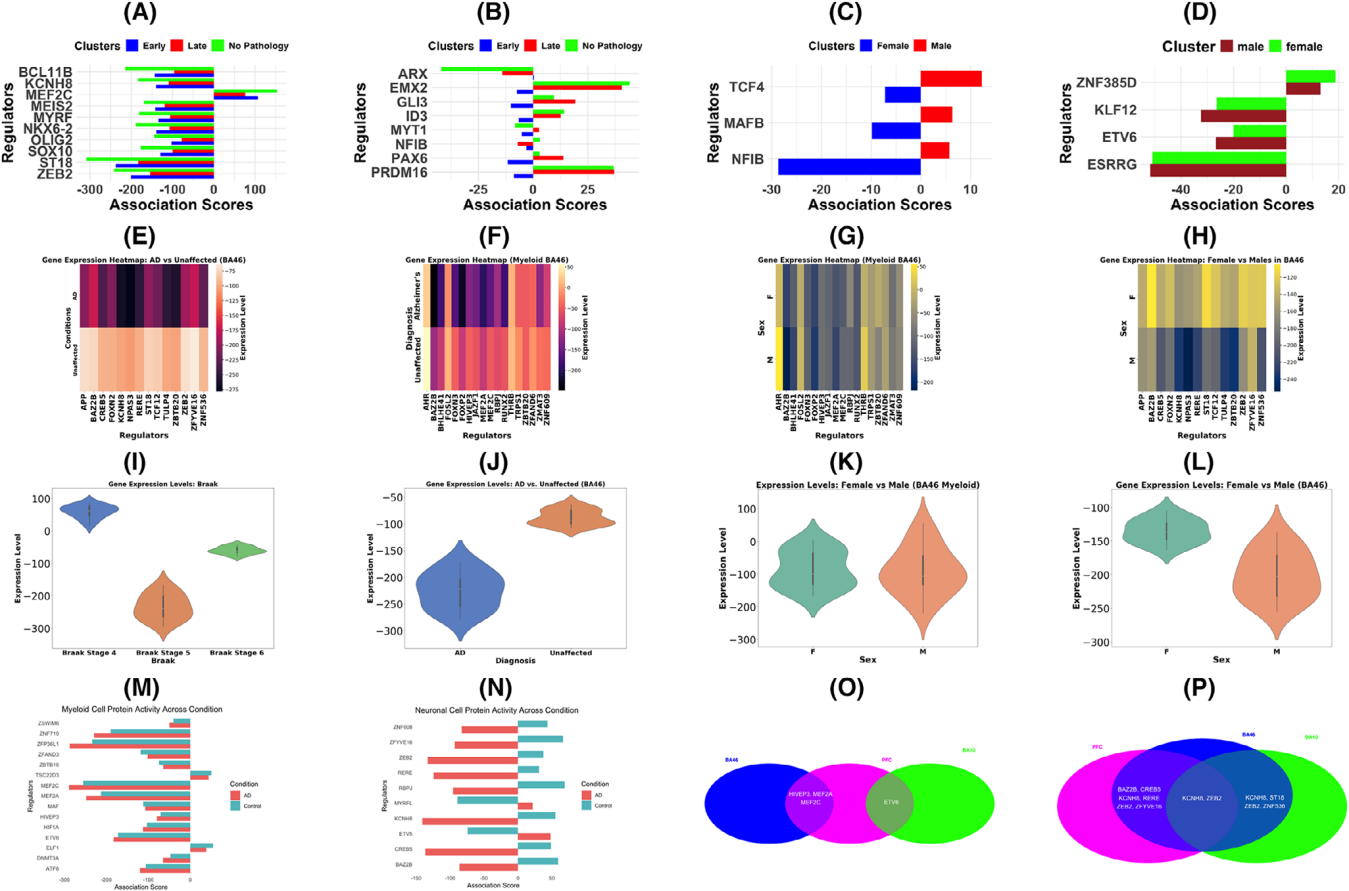
Transcriptional targets and their activity in BA10 (A–D), BA46 (E–L) and the validation (M–F). (a) Top 10 BA10 differentially expressed regulators (DERs) in neuronal cells. (b) co‐DERs with a change in expression activity between control, early and late AD in neuronal cells from BA10. (c) BA10 sex‐specific co‐DERs in neuronal cells. (d) DERs in myeloid cells from BA10. (e) BA46 top DERs in neuronal cells. (f) BA46 top DERs in myeloid cells. (g) BA46 sex‐specific DERs in myeloid cells. (h) BA46 sex‐specific DERs in neuronal cells. (i) Median DER expression levels in BA46 across Braak stages 4–6 in neuronal cells. (j) Median DER expression levels in BA46 neuronal cells for AD and unaffected conditions. (k) Sex‐specific median DER expression levels in BA46 myeloid cells. (l) Sex‐specific median DER expression levels in BA46 neuronal cells. (m), DER activities in myeloid cells showing the top regulators from the UCI validation data. (n) DER activities in neuronal cells showing the top regulators from the UCI validation data. (o) Venn diagram of the intersection of DERs identified in myeloid cells in BA46, BA10 and the validation data. (p) Venn diagram of the intersection of DERs identified in neuronal cells in BA46, BA10 and the validation data.

The key AD DERs in myeloid cells from BA10 and BA46 were MEF2A/C, HIVEP3, and ETV6 after validation (Figure 2d, f, m, o); these regulators are implicated in myeloid cell function, neuroinflammation, and AD‐related neurodegeneration.^52^, ^53^, ^54^ We found myeloid and neuronal cell‐type‐specific expression patterns of AD‐associated DERs in the PFC, and the expression of these regulators were linked with age from our analysis (see Figure S1d,e). Specific regulators in myeloid cells had mixed expressed patterns: FOSL2, THRB, AHR, BAZ2B, HIVEP3, and MEF2C, and were generally upregulated with increasing age, whilst MEF2A, ZBTB20, and RUNX2 had reduced expression. The pattern of expression of these DERs in myeloid lineage cells across Braak stage 4, 5 and 6 were mixed (Figure S1a). The median expression of DERs in neuronal cells (APP, BAZ2B, CREB5, KCNH8, ST18, ZBTB20, ZEB2, ZNF536) were reduced in AD compared to controls (Figure 2j), and were downregulated with increasing age (see Figure S1), and in individuals with higher Braak stages (> stage 4) and specific APOE alleles (e3/e4 and e4/e4) (Figure 2j and Figure S1b,c). Investigation into AD sex‐specific differences in DER expression within myeloid and neuronal cells in BA46 show that whilst the median expression levels appear lower for males compared to females (Figure 2l) in neuronal cells, the expression levels were close in myeloid cells (Figure 2k), although males have a greater range of expression variability, and higher positive values compared to females. More so, in myeloid cells, males exhibit higher expression of AHR, FOSL2, and THRB; with THRB particularly differentially expressed in males and females (Figure 2f,g and Figure S2a–da). Similarly in BA10, ESRRG, ETV6, KLF12, and ZNF385D DERs are myeloid‐specific (Figure 2d) whilst TCF4, MAFB and NFIB were differentially expressed across the sexes in neuronal cells (Figure 2c). Apart from the sex‐specific DERs, there are also distinct cell signalling patterns with sex‐specific expression, including increased activity of Autophagy‐Lysosomal Pathway (ALP) in males compared to females, and differential activation of Notch and STAT5a/b pathway between the sexes (see Figure S2a,b). Both KCNH8 and ZEB2 were the top common regulators in AD with sex‐specific differences within the PFC (Figure 2ae, h,n,p and Figure S2e,f).

It is important to mention that in BA10, PAX6 and NFIB were differentially expressed between AD and controls, and are essential in our downstream analysis due to their relevance in ocular health which is highly indicated in AD^55^ (Figure 2b). PAX6 is a master regulator of eye development and retinal patterning; a mutation to this gene can cause aniridia and other ocular or systemic issues,^55^ whilst NFIB is a downstream target of PAX6 that plays a key role in retinal development and photoreceptor differentiation.^55^, ^56^ In our study, the expression of NFIB in BA10 is coexpressed with PAX6 in control and earlyAD, but not in lateAD as seen in Figure 2b.

We observed differences in the gene regulatory networks between AD and control in BA10 and BA46, respectively, and our gene–disease interaction network analysis highlight APP as a central gene interacting with key AD regulators (see Figure S3). Validation of our results using UCI AD Multiome data^35^ show commonly expressed DERs in the PFC, BA10 and BA46 (Figure 2m–p). Further validation was done by TF enrichment analysis using ChEA3 web tool^49^ to search for overlaps between identified DERs and known TF targets from experimental sources like Genotype‐Tissue Expression (GTEx), ARCHS4 (RNA‐seq and ChIP‐seq data), and the Enrichr gene set enrichment suite. The analysis identified 1632 TF hits, including all our ARACNe‐VIPER‐inferred TFs. Amongst the top 10 ranked TFs in ChEA3 (Figure S1f) there were five BA10 neuron‐specific DERs (OLIG2, MYRF, ST18, NKX62, and SOX10) and three BA46 DERs (NPAS3, ZNF356, and ST18). ST18 and ZNF356 were common top‐ranked DERs from both UCI and ChEA3 validation results.

Thus, we have identified common DERs driving AD in specific brain areas and with distinct expression patterns in myeloid (MEF2A, MEF2C, HIVEP3, ETV6) and neuronal cells (APP, BAZ2B, MYRF, NFIB, PAX6, ST18, ZEB2, SOX10, NKX6‐2, CREB5, KCNH8, ZNF536), and in sex (TCF4, MAFB, THRB, FOSL2, ZEB2, KCNH8). The expression of these transcriptional targets was seen to decline with increasing age in neuronal cells, in e3/e4 and e4/e4 APOE alleles, and in higher Braak stages (Braak stages 5 and 6); but their expression patterns in myeloid cells appears mixed.

### 3.2 Drug screening through docking study

We applied molecular docking‐based simulations to identify the promising drugs for AD based on snRNA‐seq integrated analysis, using 20 human DERs involved in gene regulation and 3 DERs with distinct cell signalling patterns associated with sex‐specific expression and biosynthetic pathways in AD. The probable molecular interactions of drug candidates with these regulators were investigated in silico. We obtained the 3D structures of 13 DERs (APP, BAZ2B, ETV6, MEF2A, MYRF, PAX6, ST18, TCF4, THRB, ZEB2, Notch1, STAT5A, STAT5B) from the PDB using the following PDB identifiers: 3TKJ, 1AML, 2E7O, 2DAO, 1C7U, 5YHU, 1PB5, 2CUE, 2CS8, 7UBT, 6MBW, 2KWF, 1NQ0, and 2DA7. The 3D structures of the other remaining 10 (NFIB, HIVEP3, SOX10, NKX6‐2, CREB5, MAFB, FOSL2, MEF2C, KCNH8, ZNF536) were obtained from the AF model available on the UniProt server. Next, molecular docking was carried out between 23 DERs and 2200 drugs to calculate the binding affinity scores (kcal/mol) for each pair of target regulator and drugs. We used the binding energy in kcal/mol to understand and explore how well different ligands or inhibitors bind to their respective targets. Basically, ligand's affinity for a receptor protein is generally higher when the binding energy is low. So, we sorted the target regulators by their binding affinity matrix's row sums of drugs across all targets in a descending order, and the drugs according to column sums of target proteins across all drug candidates. The docking score for individual drugs against individual targets are recorded in Table S2. These scores represent the binding affinity, with more negative scores indicating stronger interactions.^57^ Drugs interacting with select target regulators (APP, ZEB2, PAX6, ETV6, ST18) with a docking score approximately equal or above −7 kcal/mol were filtered from Table S2 because this threshold is commonly considered indicative of high‐affinity interactions that warrant further investigation in in silico drug screening pipelines.^58^ After the filter was applied, we obtained 12 drugs candidates (Tables 1 and 2); six of them (Methotrexate, Lisuride, Vardenafil, Fluspirilene, Sofosbuvir, Olaparin) have been studied in AD, whilst the remaining six are yet to be studied (Tolnaftate, Alatrofloxacin, Vorapaxar, Lasmiditan, Bictegravir, and Atogepant).

**TABLE 1:** Comparable DockThor Affinity (kcal/mol) supported the reproducibility of these top‐ranked complexes, validating the reliability of Vina‐predicted affinities.

Drug	APP	PAX6	ZEB2	ETV6	ST18
Vorapaxar	−8.489	−7.715	−7.945	−7.51	−8.448
Fluspirilene	−8.251	−8.616	−7.899	−7.012	−7.611
Alatrofloxacin	−8.066	−7.291	−7.906	−7.095	−8.181
Atogepant	−7.962	−8.107	−7.563	−7.42	−7.823
Tolnaftate	−7.747	−7.913	−8.04	−7.698	−7.631
Bictegravir	−7.699	−7.795	−7.802	−7.125	−8.198
Vardenafil	−7.62	−6.914	−7.345	−7.321	−8.278
Lisuride	−7.234	−8.259	−7.409	−7.591	−7.677
Lasmiditan	−7.19	−7.181	−7.76	−7.561	−7.751
Olaparib	−7.118	−7.545	−7.42	−6.721	−7.656
Sofosbuvir	−7.027	−7.393	−7.761	−7.197	−7.902
Methotrexate	−6.788	−7.065	−6.762	−6.342	−7.165

**TABLE 2:** Blood–brain barrier analysis of selected drug candidates.

Drug	Log*P*	TPSA	MW	*pKa*	Log*D*	P‐gp Efflux	BBB permeability	logBB	logBB interpretation	DER targets > −7	Sudied in AD
Tolnaftate	4.95	12.47	307.42	8.4	3.91	1	1	0.71	Very high	18	no
Methotrexate	0.27	210.54	454.45	5.5	0.26	0	0	−2.94	Very low	18	yes
Lisuride	2.84	51.37	338.46	7.8	2.30	0	1	−0.19	Moderate	13	yes
Vardenafil	2.07	112.9	488.61	6.4	2.03	0	0	−1.22	Very low	16	yes
Fluspirilene	5.31	35.58	475.58	8.2	4.44	1	1	0.42	High	18	yes
Sofosbuvir	1.66	158.18	529.46	9.1	−0.05	0	0	−1.95	Very low	18	yes
Vorapaxar	5.63	77.52	492.59	5.9	5.62	0	1	−0.15	Moderate	15	no
Olaparib	2.35	86.37	434.47	7.2	2.13	0	1	−0.78	Very low	11	yes
Alatrofloxacin	0.90	159.65	558.52	6.1	0.88	0	0	−2.09	Very low	15	no
Lasmiditan	3.28	62.3	377.37	8	2.58	0	1	−0.29	Moderate	13	no
Bictegravir	1.63	100.87	449.39	6.8	1.54	0	0	−1.11	Very low	17	no
Atogepant	3.95	104.29	603.52	7.5	3.59	0	0	−0.80	Very low	14	no

### 3.3 Redocking and comparative scoring analysis

To enhance the reliability of docking predictions and minimise potential scoring bias inherent to a single docking engine, the top‐ranked protein–ligand complexes initially identified via AutoDock Vina were subjected to redocking using the DockThor web server (https://dockthor.lncc.br).^59^ DockThor employs an independent semiempirical scoring function and offers robust ligand flexibility handling, allowing cross‐validation of binding affinity estimates derived from Vina. The redocking analysis (see Table 1) confirmed the reproducibility of favourable binding patterns, with comparable binding affinity scores observed between DockThor and AutoDock Vina for multiple high‐ranking drug candidates across five key target proteins: APP, PAX6, ZEB2, ST18 and ETV6. For example, Vorapaxar exhibited strong binding to APP with scores of −8.489 kcal/mol (Vina) and a similarly high score from DockThor. Other drugs such as Fluspirilene, Atogepant, and Bictegravir also showed consistent ranking and affinity trends across both docking platforms. This cross‐validation reinforces the confidence in selected ligand–target pairs and reduces false positives that may arise from a single scoring algorithm. Overall, the integration of DockThor redocking enhanced the robustness of docking predictions and supports a more rigorous selection of candidate repurposable drugs for Alzheimer's disease‐related targets.

### 3.4 Evaluation of blood–brain barrier permeability for central nervous system penetration

**FIGURE 3:**
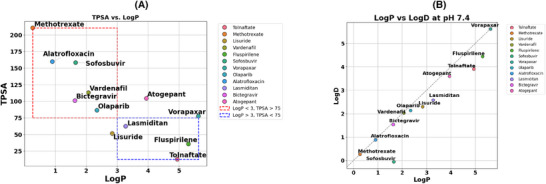
Physicochemical properties of drug candidates. (A) Topological polar surface area (TPSA) vs. Log*P*. Drugs are grouped based on TPSA and Log*P*. The red box highlights compounds with Log*P* < 3 and TPSA > 75, indicating lower membrane permeability and potentially poor central nervous system (CNS) penetration. The blue box highlights compounds with Log*P* > 3 and TPSA < 75, especially within the range Log*P* 3–4, TPSA 60–75 Å^2^. Drugs in blue box have better membrane permeability and potential CNS activity. (B) Correlation between Log*P* and Log*D* at physiological pH (7.4), showing how ionisation affects lipophilicity. The dashed line represents correlation between Log*P* and Log*D*, and data points above the line indicate increased lipophilicity under physiological conditions. Based on Log*P* and Log*D* values at pH 7.4; Vorapaxar, Fluspirilene, Tolnaftate, Atogepant, Lasmiditan, and Lisuride exhibit lipophilic characteristics (Log*P* > ∼3; Log*D* > ∼3), and have a strong potential for membrane permeability under physiological conditions. Lasmiditan and Lisuride are boarderline lipophilic.

The results of whether the selected drugs can cross the BBB effectively (see Figure 3 and Table 2) show that Tolnaftate, Lisuride, Vorapaxar, Olaparib, Lasmiditan and Fluspirilene have predicted BBB permeability. Tolnaftate and Fluspirilene both have high lipophilicity (Log*P* > 4), a logBB value greater than 0, and a relatively low topological polar surface area (TPSA < 90), which suggest they can cross the BBB effectively^60^; but they are likely P‐gp substrates (P‐gp Efflux = 1) which makes them poor CNS candidates, because of the likelihood of being actively pumped out. Moreover, Tolnaftate and Fluspirilene are in the higher safety risk space (log*P* > 3, TPSA < 75) compared to the more favourable safety risk space (Log*P* 3–4, TPSA 60–75 Å^2^) for CNS drugs.^61^ The Log*D* values also support these results, as moderately higher Log*D* values in a range (≥ 1 ≤ 3 at pH of 7.4) and logP values (≥ 2 ≤ 4) correlates with less toxicity.^60^ Vorapaxar have high lipophilicity (Log*P* > 4), but moderate logBB value; whilst Lasmiditan and Lisuride have moderate logBB, no P‐gp efflux, as well as Log*P* and TPSA values within the favourable range. As seen in Figure 3, Lasmiditan falls within the acceptable range for CNS safety and penetration. Lasmiditan also exhibits physicochemical properties comparable to well‐characterised CNS‐penetrant drugs such as risperidone (log*P* ∼2.7, TPSA ∼61.9 Å^2^, WM ∼410.5 g/mol) and donepezil (log*P* ∼3–4, MW ∼379.5 g/mol).^62^, ^63^ On the other hand, Bictegravir, Methotrexate, Alatrofloxacin, Vardenafil and Sofosbuvir have high TPSA and low LogP, indicating they are polar and less likely to cross the BBB.

### 3.5 Molecular dynamics simulation analysis to identify the optimal neuroactive drug–protein complex

To identify promising therapeutic candidates for AD, we conducted MD simulations to evaluate the binding interactions between seven drug candidates with predicted BBB permeability (Vorapaxar, Bictegravir, Tonaftate, Fluspirilene, Lisuride, Lasmiditan, and Olaparib) and five DER protei targets (ZEB2, APP, PAX6, ETV6, and ST18). The stability and binding affinity of these drug–protein complexes were assessed using key metrics including RMSD, SASA, and the number of H‐bonds. A lower protein RMSD indicates greater complex stability, higher H‐bond counts suggest stronger interactions, whilst SASA measures the surface area accessible to a solvent.^64^ In addition, we incorporated results of the critical pharmacokinetic parameters relevant to BBB permeability, including Log*P*, Log*D*, TPSA, MW, and P‐gp efflux in our interpretation.

## 4 DISCUSSION

**FIGURE 4:**
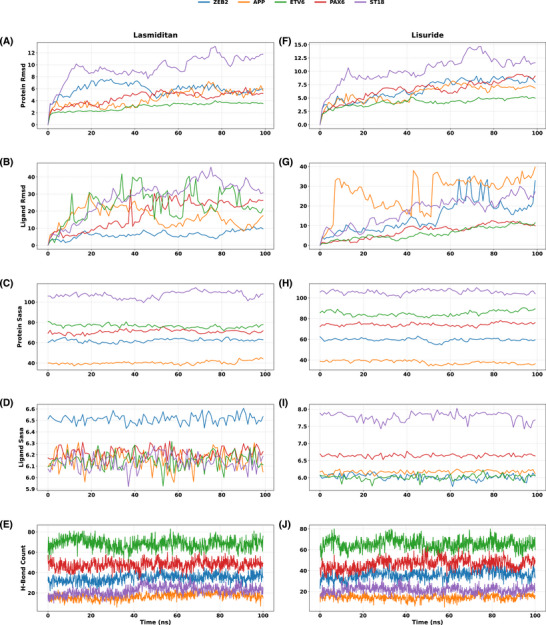
Comparative MD simulation analysis of Lasmiditan and Lisuride across five metrics in 100 ns simulation time. (a–e) represent Lasmiditan, whilst (f–j) represent Lisuride. Metrics shown include: protein root mean square deviation (RMSD) (a, f), ligand RMSD (b, g), protein solvent accessible surface area (SASA) (c, h), ligand SASA (d, i), and hydrogen bond count (e, j). Each line denotes a different protein target (APP, ETV6, PAX6, ZEB2, ST18), colour‐coded consistently across all subplots. Protein RMSD and SASA reflect the conformational stability and solvent exposure over time, respectively, whilst ligand RMSD and SASA evaluate ligand mobility and interaction interface. The hydrogen bond analysis quantifies intermolecular stability over the simulation time course.

Our MD simulation analysis (see Table S3) revealed that the Vorapaxar–APP complex (Figure S4a–e) has a relatively low protein RMSD of 4.15Å compared to other Vorapaxar–DER‐bound complexes. However, its low mean H‐bond count (15.20) and moderate BBB permeability (logBB: –0.15) limits its potential as a strong candidate. Bictegravir–ETV6 (Figure S4f–j) show the lowest protein RMSD amongst all Bictegravir‐DER‐bound complexes (3.34 Å) and a relatively high number of H‐bonds which indicates good stability and binding affinity; however, its very low BBB permeability (logBB: –1.105) makes it a suboptimal neuroactive agent. A similar pattern is observed with Olaparib–ETV6 (Figure S5a–e: RMSD: 3.25 Å, H‐bonds: 71.20) which exhibits poor BBB permeability (logBB: –0.78) despite favourable binding characteristics. In contrast, Tonaftate‐ETV6 (Figure S6a–e: RMSD 3.13 Å, 65.94 H‐bonds, logBB 0.71), Fluspirilene‐ETV6 (Figure S6f–j: RMSD 3.26 Å, 68.39 H‐bonds, logBB 0.42), Lasmiditan‐ETV6 (Figure 4a–e: RMSD 2.98 Å, 68.38 H‐bonds, logBB −0.29), and Lisuride‐ETV6 (Figure 4f–j: RMSD 4.17 Å, 65.57 H‐bonds, logBB −0.19) have more favourable combination of stability, strong interactions, and moderate to high predicted BBB permeability. Nevertheless, only Lasmiditan and Lisuride have a balance between pharmacodynamic and pharmacokinetic properties because both Tolnaftate and Fluspirilene are potential P‐gp substrates (P‐gp+) which means they are likely to be expelled from the brain after BBB entry.

Whilst Lisuride has previously been studied in AD in a randomised double‐blind trial,^65^ which showed no strong statistically significant effects but some improvement in verbal learning, Lasmiditan represents a candidate with no prior AD association. There are no record of a clinical trial of Lasmiditan specifically for AD in ClinicalTrials.gov, and no published data specifically evaluating its transcriptional regulation or binding interaction with AD‐relevant transcription factors. Lasmiditan has optimal passive diffusion thresholds (Log*P* > 3, TPSA < 75)^61^ and the interaction with ETV6 (Figure 4a–e) shows exceptional stability by having the lowest protein RMSD (2.98 ± 0.69 Å) amongst all drug–DER protein complexes examined, and a robust hydrogen bonding (68.38 ± 5.22) which indicates minimal structural disruption and strong intermolecular interactions. Nonetheless, Lasmiditan‐ETV6 complex has moderate BBB permeability (LogBB: −0.29) compared to Tonaftate‐ETV6 (LogBB: 0.71), Fluspirilene (LogBB: 0.42) and Lisuride (LogBB: −0.19). Also, the SASA analysis show that Tonaftate, Lisuride and Fluspirilene have efficient ligand burial (ligand SASA: 5.70 Å^2^, 6.01 Å^2^ and 7.79 Å^2^, respectively) with moderate protein surface exposure (82.89 Å^2^, 85.03 Å^2^, 87.00 Å^2^) compared to Lasmiditan (Protein SASA 87.00 Å^2^, Ligand SASA 8.68 Å^2^), which suggest that they have strong and compact binding than Lasmiditan. However, the slightly higher ligand exposure to solvent for Lasmiditan‐ETV6 is compensated by its strong hydrogen bonding interactions.

Considering all metrics together, our integrative analysis combining snRNA‐seq, molecular docking, dynamics simulations, and pharmacokinetic profiling identifies Lasmiditan as the most promising drug candidate. As shown in our analysis, ETV6 is a viable therapeutic target due to its ability to form stable and strongly interacting complexes across multiple drugs. It is potentially druggable, as seen in the structural analysis which reveals the predicted pocket sites and drug scores > 0.7 (see Table S4) of identified DERs. The favourable druggability score (> 0.7) and predicted ligand pocket suggest ETV6 is targetable without off‐target toxicity. ETV6 is known for its role in monocyte differentiation and has been identified as a marker for microglial cells^66^, ^67^; which are the primary immune cells of the brain,^68^ that are highly important in understanding AD progression.^66^, ^69^ Although ETV6 have not been largely associated with AD; it is involved in immune regulation^66^, ^67^, ^70^ and our analysis shows that it has altered expression patterns in BA10 myeloid‐specific gene expression in AD. Based on our results, Lasmiditan–ETV6 complex appears to be the best drug candidate based on its high H‐bonds, acceptable protein RMSD, and optimal passive diffusion thresholds. We acknowledge that relying on hard cutoffs may not fully capture the multidimensional nature of drug efficacy and pharmacokinetics;^71^ therefore, our conclusions are based on a holistic integration of all metrics.

In its current use, Lasmiditan is approved for acute migraine treatment, and administered as a single dose.^72^ Although Lasmiditan is FDA‐approved for acute migraine, its CNS exposure, oral bioavailability, and non‐vasoconstrictive profile support its potential in neurodegenerative contexts.^72^, ^73^ It is uncertain whether it can be taken chronically, and at an effective dose with respect to ETV6 mediated effects, which warrants further future exploration. Lasmiditan is a 5‐HT₁F receptor agonist, that inhibits the release of pro‐inflammatory neuropeptides like calcitonin gene‐related peptide (CGRP), and modulates trigeminal pain pathways without causing vasoconstriction.^72^, ^74^ Its receptor‐mediated mechanism may influence transcriptional regulation through downstream effects on CGRP and glutamate,^74^, ^75^ though its role in modulating protein–protein interactions is unknown and warrants biochemical validation. The existing AD therapies such as Lecanemab and Donanemab are anti‐amyloid therapies that target amyloid‐β (Aβ) aggregates to promote plaque clearance, but carry a risk of amyloid‐related imaging abnormalities (ARIA).^76^, ^77^ Whilst Lasmiditan has no reported ARIA risk, some of its known side effects include dizziness, drowsiness and fatigue,^76^, ^77^ so it may offer an alternative to compliment current therapies which do not target non‐amyloid pathways, after further preclinical safety validation proves successful.

Although both Lasmiditan and Lisuride exhibited strong interaction profiles with ETV6, Lasmiditan had the lowest RMSD (2.98 Å) and highest H‐bond count, indicating a more stable binding. Moreover, Lisuride is an older parenteral dopamine agonist for Parkinson's disease,^78^ that is not approved in the United States^79^; and its side effects (nausea, dizziness, hallucinations) and ergot‐related risks^80^ (cardiac/pulmonary fibrosis) limit its tolerability in AD patients and make it unsuitable for chronic use. The identification of Lasmiditan along with six previously investigated drugs (see Table 2) in AD, underlies the usability and rigor of our approach. It is important to note that our conclusions are not based on individual cutoffs alone, but on integrative ranking across multiple metrics (RMSD, H‐bond count, logBB, drug score, etc). We emphasise that whilst repurposing may reduce early‐phase failure rates and provide increased efficacy, it still faces challenges in efficacy, dosing, drug–drug interactions, and regulatory approval^81^, ^82^ which are beyond the scope of the current study. Indeed, our work serves as a first step and is not a substitute for traditional drug development. Our findings will provide a strong foundation for future experimental and clinical direction to evaluate behaviour, plasma biomarkers, and test whether chronic Lasmiditan administration modifies the disease progression.

## AUTHOR CONTRIBUTIONS

This study was designed by Martin Nwadiugwu. Martin Nwadiugwu, Md Selim Reza and Hongwen Deng were involved in conceptualisation, methodology, directing and coordinating the research. Martin Nwadiugwu performed snRNA‐seq analysis, molecular dynamics simulation and pharmacokinetic profilling. Md Selim Reza performed molecular docking analysis. Boluwatife Afolabi, Demetrius M. Maraganore, Hui Shen and Hongwen Deng reviewed and edited the manuscript. Martin Nwadiugwu wrote the manuscript.

## CONFLICT OF INTEREST STATEMENT

The authors declare no conflicts of interest.

## ETHICS APPROVAL

Not applicable.

## CODE AVAILABILITY

Code to reproduce the analyses can be accessed via: https://github.com/martintony4all/singlecellAD and https://github.com/martintony4all/MolecularDynamicsSimulation

## Supporting information



Supporting information

Supporting information

Supporting information

Supporting information

Supporting information

Supporting information

## Data Availability

Demographic, clinical and snRNA‐seq data for ROSMAP and UCI AD data can be accessed via the AD Knowledge Portal (adknowledgeportal.org) under synapse ID “syn18485175”, and “syn22079621,” respectively. The Demographic, clinical and snRNA‐seq data from Anderson et al.^34^ study can be accessed in the Gene Expression Omnibus (GEO) database under accession number “GSE214637”. All other relevant data supporting the key findings are available within the Supporting information files.
