# Supplementary material for: Integrative snRNA‐seq, molecular docking and dynamics simulations identifies Lasmiditan as drug candidate for Alzheimer's disease
Source: Clin Transl Med. 2025 Aug 15;15(8):e70443. doi: 10.1002/ctm2.70443 (PMC12356828; doi:10.1002/ctm2.70443)
Supplement: Supplementary file 1 — Supporting information [file CTM2-15-e70443-s006.docx]

**Sex-Specific Differences and Expression Patterns of AD Regulators**

We observed the sex-specific differences among males and females when analyzing the combined gene regulatory network of BA10 and BA46 using IPA software (Figures S2a, S2b). As can be seen, THRB was upregulated in males compared to females (Figure S2a and S2b), while FOSL2 is also found to be expressed differently in AD compared to controls in Figures S2c and S2d. In terms of cell signaling patterns, the Autophagy–Lysosomal Pathway (ALP) is activated in males compared to females (Figure S2a, S2b), which suggest males may have a heightened capacity for degrading misfolded or aggregated proteins, because ALP deficits results in protein aggregation which is central to AD pathology^1^. Meanwhile, the Notch and STAT5a/b pathways are activated in females but inhibited in males, suggesting sex-based differences in vascular-related cognitive impairments^2,3^. The Notch signaling pathway is associated with genetic forms of vascular and Alzheimer's dementia^3^ while the STAT5a/b pathway influences sex bias in vascular disease^2^. These findings show that FOSL2 and THRB, along with ALP, Notch, and STAT5a/b signaling pathways, may drive sex differences in AD susceptibility and progression.

**Regulatory Expression Profiles in BA46 Across Aging and Clinicopathological Conditions**

In BA46, neuron-specific DERs (including APP, BAZ2B, CREB5, FOXN2, KCNH8, NPAS3, RERE, ST18, TCF12, TULPA, ZBTB20, ZEB2, ZFYVE16, and ZNF536) exhibited declining expression with increasing age (66-92 years) in neuronal cells, but mixed expression in myeloid cell subsets (**Figures S1D, S1E**), suggesting a potential reduction in neuronal activity in AD pathogenesis. This decline was also seen in individuals with APOE E3/E4 or E4/E4 alleles and advanced tau pathology (Braak stage 5/6) compared to those with protective genotypes or earlier Braak stages (**Figures S1A, S1B, S1E**), indicating a potential link between increasing age ^4,5^, tau pathology, APOE genotype, and neuronal regulatory dynamics relevant to AD.^6,7^

**Gene-Disease Interaction and DER Regulatory Networks in BA46 and BA10**

Integrated analysis of BA46 and BA10 gene regulatory networks (GRNs) using IPA revealed distinct differences between AD and control states (**Figures S2C-S2K**). Analysis of myeloid cells revealed sex-specific DER expression patterns in BA46 (**Figures S2A, S2B**). BA46 males exhibited higher expression of THRB, as GRN analysis show increased THRB in BA46 males (**Figure S2A**), compared to females (**Figure S2B**). We also notice activation of the Autophagy–Lysosomal Pathway (ALP) in males compared to females, and the inactivation of NOTCH and STAT5A/B cell signaling pathways in males which are activated in females. Functional analysis from previous studies suggests that increased ALP activity in males, may reflect greater capacity for protein degradation^8^, and increased Notch/STAT5a/b activity in females, is possibly linked to sex-based differences in vascular cognitive impairment. ^2,3^ In the combined BA10/BA46 AD GRN (**Figure S2E**), downregulation of ZEB2, a key regulator, was associated with inhibition of OLIG1 and OLIG2, whereas ZEB2 upregulation in the control GRN activated these targets (**Figure S2F**). BA-specific GRN analysis showed PAX6 and NFIB in BA10 GRNs (**Figures S2G, S2H, S2I**) to be differentially expressed in early, late AD and in controls. Furthermore, within BA46, the AD GRN (**Figure S2K**) exhibited increased activation of DERs and miRNAs (miR-205, miR-92a-3p, miR-25) compared to the control (**Figure S2J**), which showed inactivation of ZEB2, TP53, CREB5, BAZ2B, ZFYVE16, and RERE, leading to repression of JUN, GSC, miR-218, miR-630, and miR-221. These observations indicate that BA46 may be undergoing a dysregulated cellular environment in AD,^9–13^ which could help explain AD vulnerability in specific brain area.^11^

A comprehensive gene interaction network (**Figure S3**) shows APP as a central node in AD-related regulatory mechanisms and highlighted its central role, linking it to memory deficits, epilepsy, and nerve degeneration^8^ via interactions with ZEB2, APOE, ZFYVE16, and TP53. The connection of KCNH8 and ZEB2 to critical neurological outcomes within this network emphasizes the importance of these regulators in AD pathogenesis.

**Validation Using ChEA3 Web Tool**

To further validate our findings, we utilized the ChIP-X Enrichment Analysis Version 3 (ChEA3) tool,^14^ which integrates data from diverse libraries (GTEx, ARCHS4, Enrichr) to identify and rank transcription factors (TFs) from experimental studies based on overlaps with differentially expressed regulators (DERs) in our study. Using our combined DERs from all brain areas as input, ChEA3 identified 1,632 TFs, and five of the top 10 TFs from ChEA3 mean ranks were BA10 DERs (OLIG2, MYRF, ST18, NKX6-2, SOX10) (**see Figure S1F**). Furthermore, NPAS3, ZNF536, and ST18, identified as top neuron-specific DERs in BA46 (Figure S1F), were also present in the ChEA3 top-ranked TFs which further supports our findings. ZNF536 and ST18 were common regulators in BA10, BA46 and ChEA3 analysis.

**Supplementary Tables**

Supplemental Table 1 (Table S1): List of approved FDA drugs from DrugBank database

Supplemental Table 2 (Table S2): The docking score for individual drugs

Supplemental Table 3 (Table S3): MD Analysis of drug candidates

Supplemental Table 4 (Table S4): Druggable binding pockets in the top-ranked DERs

**References**

1. Martini-Stoica, H., Xu, Y., Ballabio, A. & Zheng, H. The Autophagy-Lysosomal Pathway in Neurodegeneration: A TFEB Perspective. *Trends in Neurosciences* vol. 39 Preprint at https://doi.org/10.1016/j.tins.2016.02.002 (2016).

2. Sehgal, P. B., Yang, Y. M., Yuan, H. & Miller, E. J. STAT5a/b contribute to sex bias in vascular disease: A neuroendocrine perspective. *JAK-STAT* vol. 4 Preprint at https://doi.org/10.1080/21623996.2015.1090658 (2015).

3. Kapoor, A. & Nation, D. A. Role of Notch signaling in neurovascular aging and Alzheimer’s disease. *Semin Cell Dev Biol* **116**, (2021).

4. Hermann, D. M., Peruzzotti-Jametti, L., Giebel, B. & Pluchino, S. Extracellular vesicles set the stage for brain plasticity and recovery by multimodal signalling. *Brain* vol. 147 Preprint at https://doi.org/10.1093/brain/awad332 (2024).

5. Bishop, N. A., Lu, T. & Yankner, B. A. Neural mechanisms of ageing and cognitive decline. *Nature* vol. 464 Preprint at https://doi.org/10.1038/nature08983 (2010).

6. Karch, C. M. & Goate, A. M. Alzheimer’s disease risk genes and mechanisms of disease pathogenesis. *Biol Psychiatry* **77**, 43–51 (2015).

7. Yamazaki, Y., Zhao, N., Caulfield, T. R., Liu, C.-C. & Bu, G. Apolipoprotein E and Alzheimer disease: pathobiology and targeting strategies. *Nat Rev Neurol* **15**, 501–518 (2019).

8. Martini-Stoica, H., Xu, Y., Ballabio, A. & Zheng, H. The Autophagy-Lysosomal Pathway in Neurodegeneration: A TFEB Perspective. *Trends in Neurosciences* vol. 39 Preprint at https://doi.org/10.1016/j.tins.2016.02.002 (2016).

9. Matarin, M. *et al.* A Genome-wide gene-expression analysis and database in transgenic mice during development of amyloid or tau pathology. *Cell Rep* **10**, (2015).

10. Wang, M. *et al.* Integrative network analysis of nineteen brain regions identifies molecular signatures and networks underlying selective regional vulnerability to Alzheimer’s disease. *Genome Med* **8**, (2016).

11. Iaccarino, L. *et al.* Local and distant relationships between amyloid, tau and neurodegeneration in Alzheimer’s Disease. *Neuroimage Clin* **17**, (2018).

12. Marcus, D. L. *et al.* *Quantitative Neuronal C-Fos and c-Jun Expression in Alzheimer’s Disease*. (1998).

13. De Strooper, B. & Karran, E. The Cellular Phase of Alzheimer’s Disease. *Cell* vol. 164 Preprint at https://doi.org/10.1016/j.cell.2015.12.056 (2016).

14. Keenan, A. B. *et al.* ChEA3: transcription factor enrichment analysis by orthogonal omics integration. *Nucleic Acids Res* **47**, W212–W224 (2019).
