# Supplementary material for: Integrative snRNA‐seq, molecular docking and dynamics simulations identifies Lasmiditan as drug candidate for Alzheimer's disease
Source: Clin Transl Med. 2025 Aug 15;15(8):e70443. doi: 10.1002/ctm2.70443 (PMC12356828; doi:10.1002/ctm2.70443)
Supplement: Supplementary file 6 — Supporting information [file CTM2-15-e70443-s005.pdf]

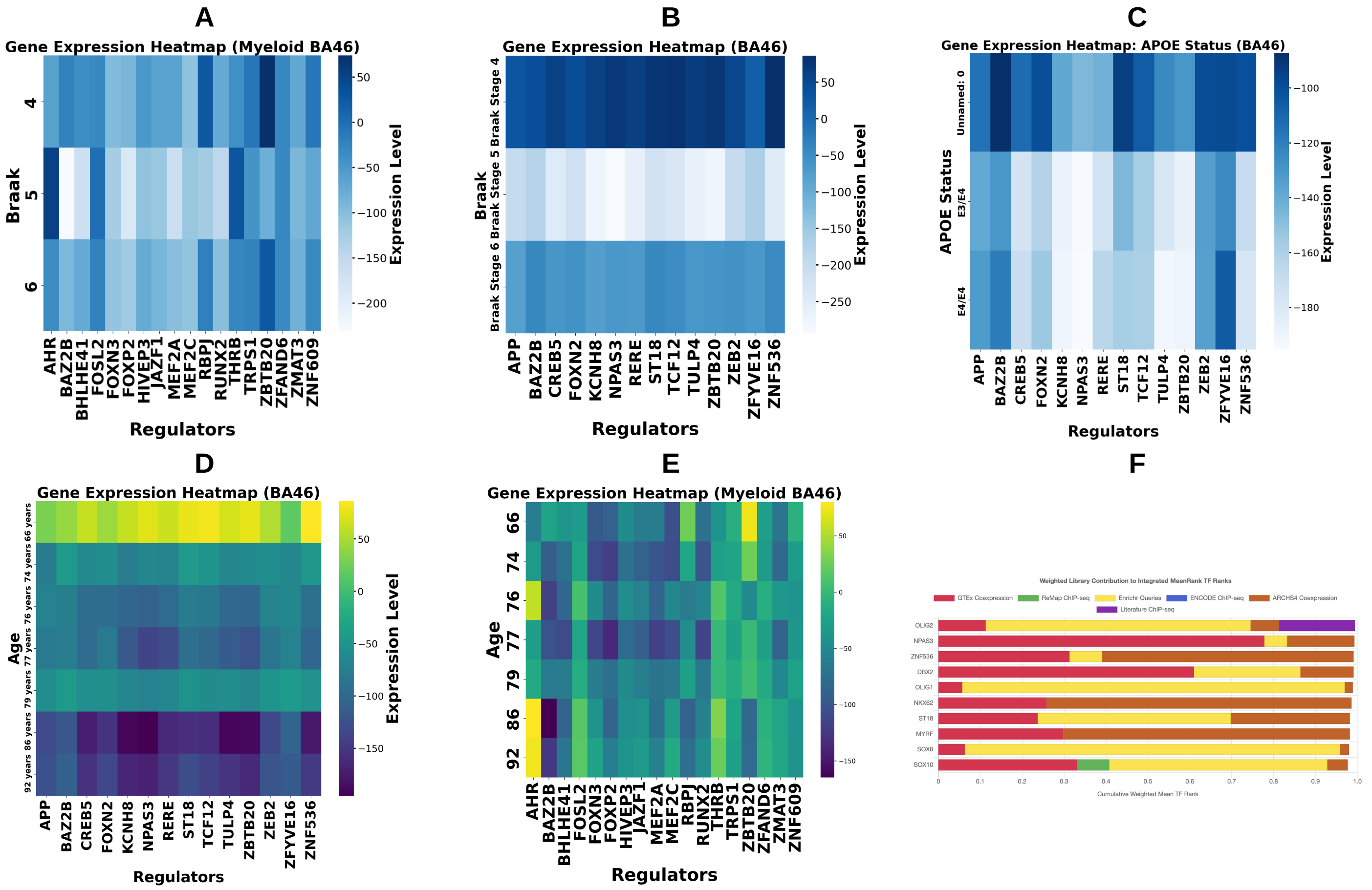

Figure S1: Differentially expressed regulators (DERs) and their activity in BA46 across braak stage (A-B), APOE alele (C), age (D-E) and the ChEA3 validation (F). S1a, Heatmap illustrating DERs in myeloid cells across Braak Stages 4, 5 and 6 in BA46. S1b, Heatmap illustrating DERs in neuronal cells across Braak Stages 4, 5 and 6 in BA46. S1c, DERs in neuronal cells across APOE major allelic variants (ε3 and ε4) and individuals without these allelic variants in BA46. S1d, DERs across age (66, 74, 76, 77, 79, 86, and 92 years) in BA46 myeloid cells. S1e, DERs across age (66, 74, 76, 77, 79, 86, and 92 years) in BA46 neuronal cells. S1f, Validation results of weighted contribution of various ChEA3 libraries to BA46/BA10 common DERs.

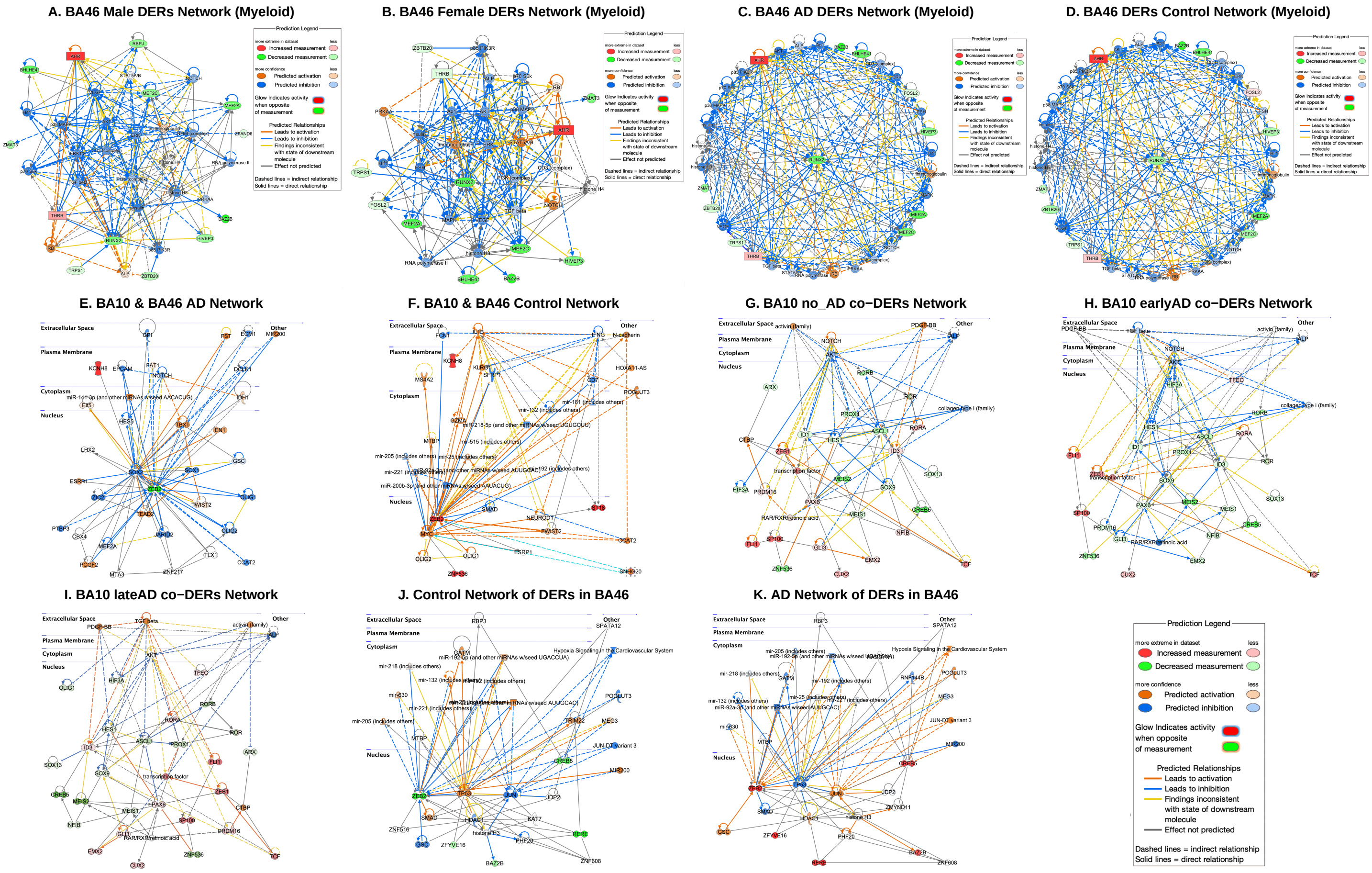

Figure S2: BA10, BA46, and sex-specific DER networks in myeloid (A-D) and neuronal cells (E-K). S2a, DER network in males from BA46 myeloid cells showing activated ALP, and inhibited Notch and STAT5a/b cell signaling pathway. S2b, DER network in females from BA46 myeloid cells showing reduced ALP activation, and increased Notch and STAT5a/b activity. S2c, AD DER network in BA46 myeloid cells showing downregulated FOSL2. S2d, DER network in controls from BA46 myeloid cells showing upregulated FOSL2. S2e, AD interaction network of DERs in both BA10 and BA46. S2f, Interaction network of DERs in controls in BA10 and BA46. S2g, BA10 co-DER interaction network in controls. S2h, BA10 co-DER interaction network in early AD. S2i, BA10 co-DER interaction network in late AD. S2j, BA46 DER interaction network in controls. S2k, AD DER interaction network in BA46.

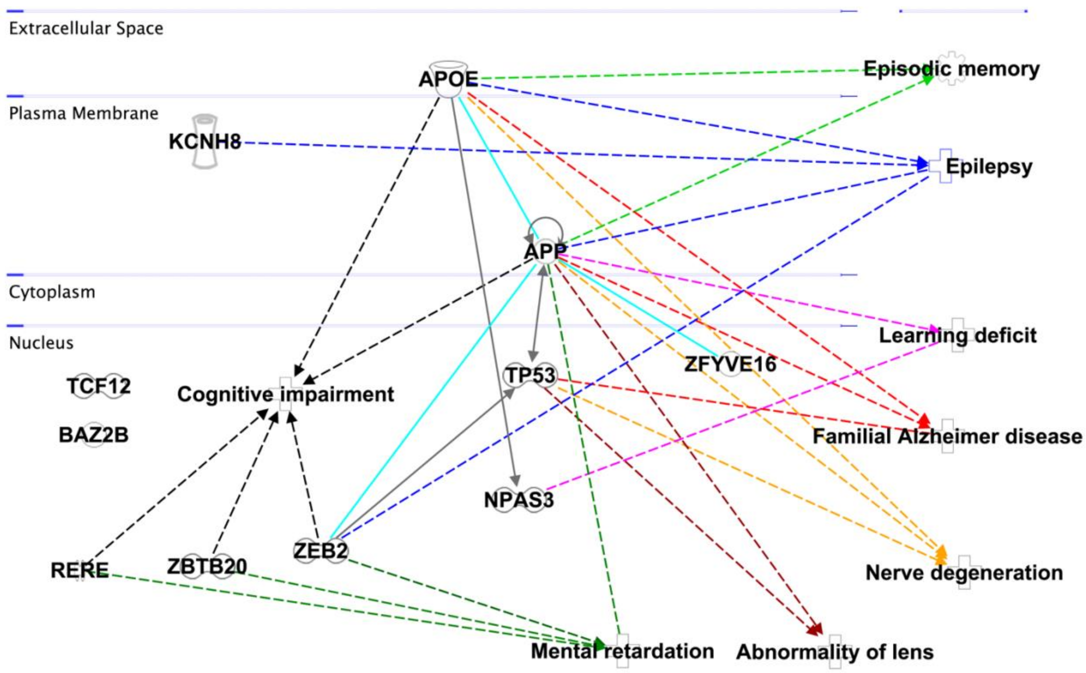

Figure S3: Gene-Disease Network showing association between Key DERs and AD Pathology.

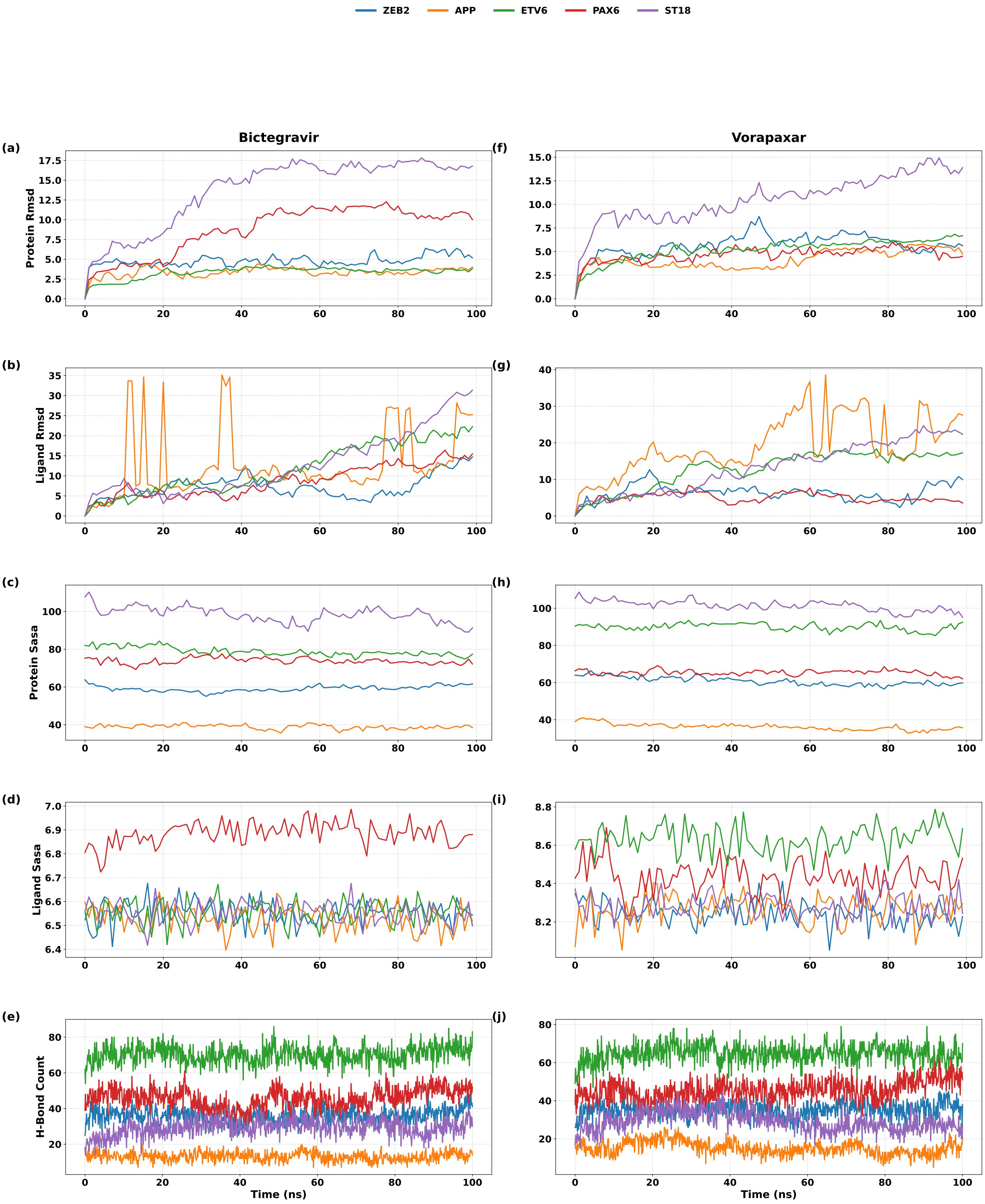

Figure S4: Comparative molecular dynamics analysis of Bictegravir and Vorapaxar across five metrics in 100 ns. Figures a–e represents Bictegravir, while f–j represent Vorapaxar. Metrics shown include Protein RMSD (a, f), Ligand RMSD (b, g), Protein SASA (c, h), Ligand SASA (d, i), and Hydrogen Bond Count (e, j). Each line denotes a different protein target (APP, ETV6, PAX6, ZEB2, ST18), color-coded consistently across all subplots. Protein RMSD and SASA reflect the conformational stability and solvent exposure over time respectively, while ligand RMSD and SASA evaluate ligand mobility and interaction interface. The hydrogen bond analysis quantifies intermolecular stability over the simulation time course.

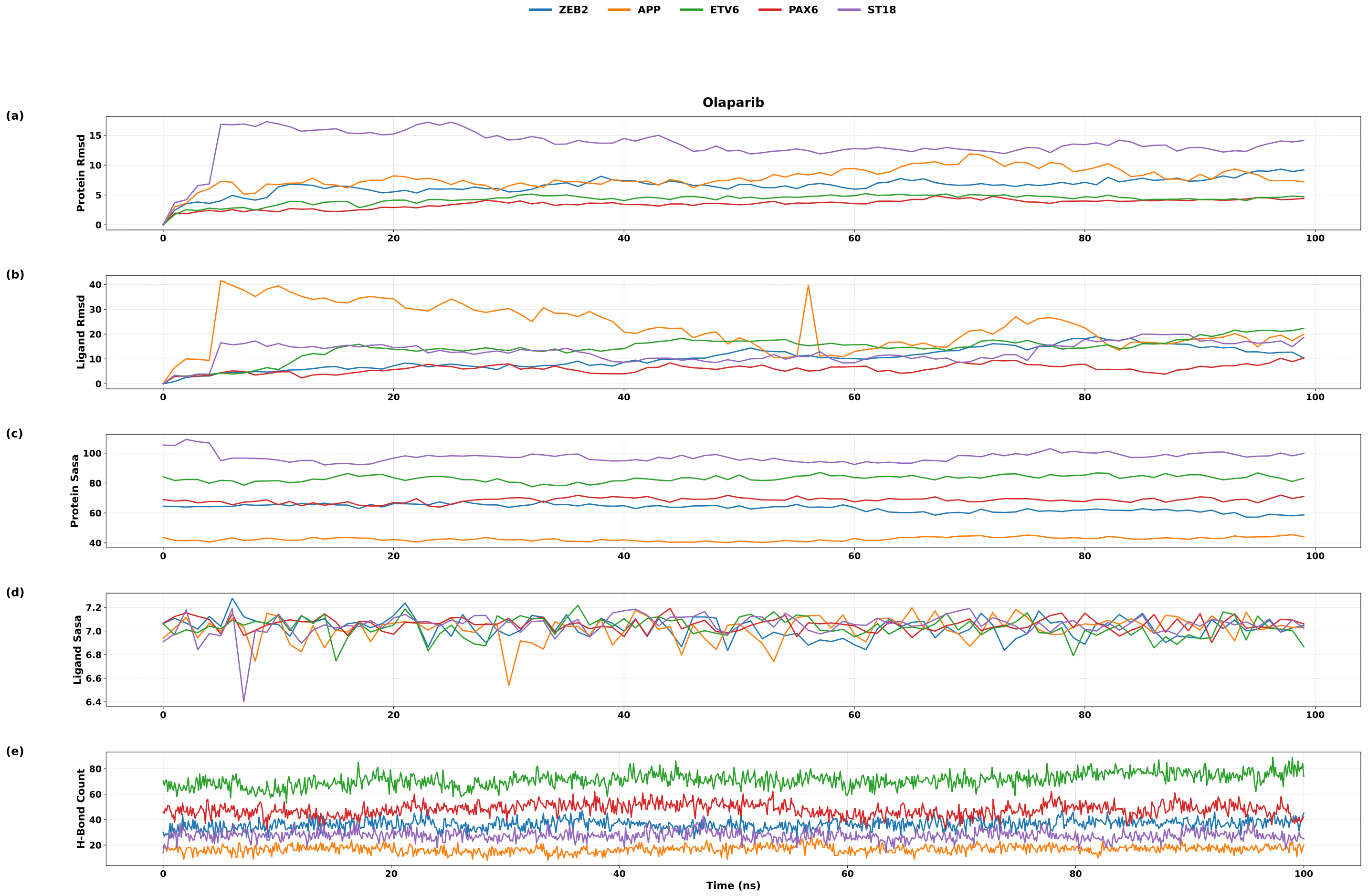

Figure S5: Comparative molecular dynamics analysis of Olaparib across five metrics in 100 ns. Metrics shown include Protein RMSD (a), Ligand RMSD (b), Protein SASA (c), Ligand SASA (d), and Hydrogen Bond Count (e). Each line denotes a different protein target (APP, ETV6, PAX6, ZEB2, ST18), color-coded consistently across all subplots. Protein RMSD and SASA reflect the conformational stability and solvent exposure over time respectively, while ligand RMSD and SASA evaluate ligand mobility and interaction interface. The hydrogen bond analysis quantifies intermolecular stability over the simulation time course.

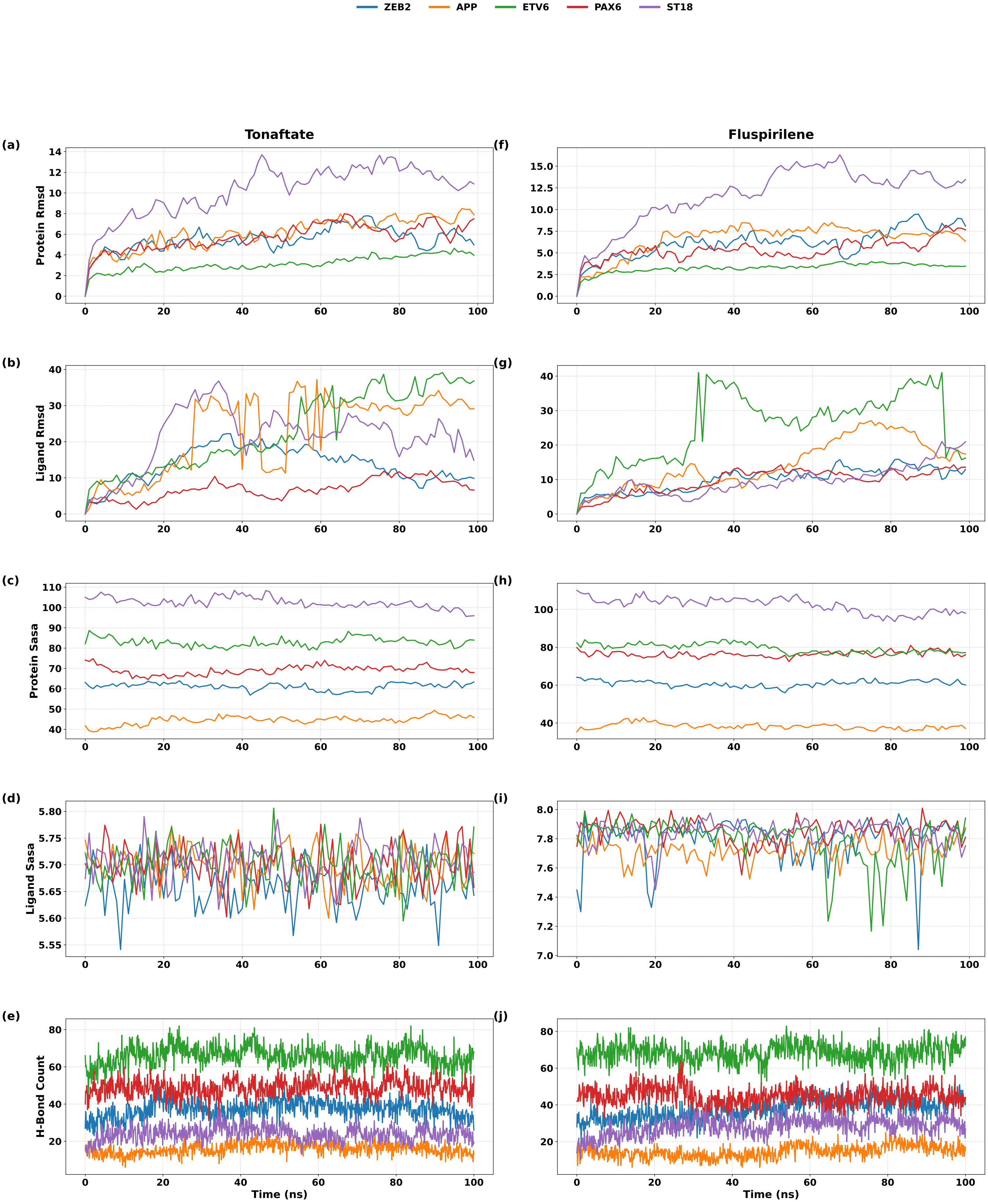

Figure S6: Comparative molecular dynamics analysis of Tonaftate and Fluspirilene across five metrics in 100 ns. Figures a–e represents Tonaftate, while f–j represent Fluspirilene. Metrics shown include: Protein RMSD (a, f), Ligand RMSD (b, g), Protein SASA (c, h), Ligand SASA (d, i), and Hydrogen Bond Count (e, j). Each line denotes a different protein target (APP, ETV6, PAX6, ZEB2, ST18), color-coded consistently across all subplots. Protein RMSD and SASA reflect the conformational stability and solvent exposure over time respectively, while ligand RMSD and SASA evaluate ligand mobility and interaction interface. The hydrogen bond analysis quantifies intermolecular stability over the simulation time course.
